# Hyperthermophilic L-Asparaginase from *Thermococcus sibiricus* and Its Double Mutant with Increased Activity: Insights into Substrate Specificity and Structure

**DOI:** 10.3390/ijms26125437

**Published:** 2025-06-06

**Authors:** Maria V. Dumina, Dmitry D. Zhdanov, Alexander V. Veselovsky, Marina V. Pokrovskaya, Svetlana S. Aleksandrova, Mikhail E. Minyaev, Larisa A. Varfolomeeva, Ilya O. Matyuta, Konstantin M. Boyko, Alexander A. Zhgun

**Affiliations:** 1 Federal Research Center “Fundamentals of Biotechnology” of the Russian Academy of Sciences, Moscow 117312, Russia; 2 Institute of Biomedical Chemistry, Moscow 119121, Russiaveselov@ibmh.msk.su (A.V.V.); ivan1190@yandex.ru (M.V.P.);; 3 N.D. Zelinsky Institute of Organic Chemistry Russian Academy of Sciences, Moscow 119071, Russia; 4 Moscow Center for Advanced Studies, Moscow 123592, Russia

**Keywords:** L-asparaginase, hyperthermophilic enzyme, substrate specificity, stereoselectivity, thermo-L-asparaginase structure

## Abstract

L-asparaginase (L-ASNase) is a key industrial enzyme significant for cancer therapy and the food industry for reducing dietary acrylamide. The hyperthermophilic L-ASNase from *Thermococcus sibiricus* (TsAI) was previously shown to exhibit high activity and thermostability and is promising for biotechnology. To gain insights into structure-functional relationships of TsAI, determination of the substrate specificity, kinetic parameters, structural characterization, and molecular docking were performed. TsAI characteristics were compared with the TsAI^D54G/T56Q^ mutant, which exhibited increased activity after a double mutation in the substrate-binding region. TsAI and TsAI^D54G/T56Q^ were found to display high activity towards D-asparagine—62% and 21% of L-asparaginase activity, respectively—and low L-glutaminase coactivity of ~5%. Restoring the mesophilic-like triad GSQ in the mutant resulted in a two-fold increase in activity towards L-asparagine compared with TsAI. Crystal structures of TsAI forms solved at 1.9 Å resolution revealed that double mesophilic-like mutation increased the flexibility of the loop M51-L57, located in close proximity to the active site. Structural superposition and mutational analysis indicate that mobility of this loop is essential for the activity of thermo-ASNases. Molecular docking, without taking into account the temperature factor, showed that, in contrast to L-asparagine interaction, D-asparagine orientation in the TsAI and TsAI^D54G/T56Q^ active sites is similar and not optimal for catalysis. Under real conditions, high temperatures can induce structural changes that reduce L-ASNase discrimination towards D-asparagine. Overall, the obtained structural and biochemical data provide a basis for a more detailed understanding of thermo-ASNase functioning and possibilities to engineer improved variants for future biotechnological application.

## 1. Introduction

L-asparaginase (L-ASNase (EC 3.5.1.1; or L-asparagine amidohydrolase)) occupies a significant niche among key industrial enzymes due to its therapeutic applications [1]. L-ASNase exerts its anticancer activity by hydrolyzing L-asparagine (L-Asn) in the bloodstream to L-aspartic acid and ammonia. This activity targets tumors with low levels of asparagine synthetase expression, which have a strict requirement for extracellular L-Asn for survival [2]. Currently, only type II L-ASNases from the mesophilic bacteria *Escherichia coli* (EcAII) and *Dickeya dadanti* (formerly known as *Erwinia chrysanthemi* (ErAII)) are approved for clinical use [3,4].

A novel, non-medical, but health-related area of L-ASNase application is prevention of carcinogenic by-product formation in fried and baked foods [5]. Asparagine reacts with reducing sugars during the initial stages of the Maillard reaction, which occurs at temperatures above 120 °C. This reaction generates a number of carcinogenic compounds, including acrylamide [6]. L-ASNase effectively prevents the formation of hazardous derivatives in high-temperature food technologies by hydrolyzing substrate for their synthesis [7].

For food industry applications, L-ASNase variants with high activity at elevated temperatures and thermostability are required. Among L-ASNases characterized to date, thermophile- and hyperthermophile-derived enzymes exhibit unique characteristics at high temperatures [8]. A promising type I-like thermo-ASNase from the archaea *Thermococcus sibiricus* (TsAI) was previously described [9]. The enzyme is characterized by its high specific activity at 90 °C and a relatively low K_m_ value compared with other L-ASNases of hyperthermophilic origin [8].

To improve TsAI properties, a mutant with conjoint substitution of two residues adjacent to the substrate-binding S55 was engineered based on multiple sequence alignment and homologous modeling [10]. All types of L-ASNases contain several highly conserved amino acid residues [8]. T12, Y25, S58, Q59, T89, D90, K162, and E283 were found to be essential for the catalysis of the type II L-ASNase from *E.coli*, a representative member of a large family of L-ASNases [11,12,13,14]. T12, Y25, T89, and K162 are directly involved in enzyme catalysis, while S58, Q59, D90, and E283 are responsible for substrate binding [15]. The same residues occupy the corresponding positions of the type I-like thermo-ASNases from *Thermococcus* sp., including TsAI. The exception is the residue that corresponds to Q59 in EcAII, which is substituted by threonine in thermo-ASNases. The residue corresponding to G57 of EcAII, adjacent to the substrate binding S58, in thermo-ASNases is also replaced by the highly conserved aspartate. G57 in EcAII was found to affect substrate specificity of the enzyme [16,17]. The mesophilic combination of highly conserved residues involved in catalysis and substrate-enzyme interaction was restored in TsAI by the double mutation D54G/T56Q (TsAI^D54G/T56Q^) that resulted in a two-fold increase in activity [10]. However, structural changes occurred, and data underlying the drastic increase in activity of the mutant form were not available. In this study, the crystal structures of the TsAI parent enzyme and its mutant TsAI^D54G/T56Q^ “mimicking” substrate–enzyme interaction of the type II L-ASNase EcAII were obtained. Comparison of the activity towards L-ASNase substrates and kinetic parameters was performed. To study differences in interaction with enantiomers of asparagine, molecular docking was applied for TsAI and its mutant TsAI^D54G/T56Q^. The results of the study provide more details about the structure and specificity of thermo-ASNases, which are promising for use in biotechnology, in particular, in the food industry.

## 2. Results

### 2.1. Comparison of the Main Enzymatic Properties of TsAI Forms

#### 2.1.1. Substrate Specificity

**Figure 1:**
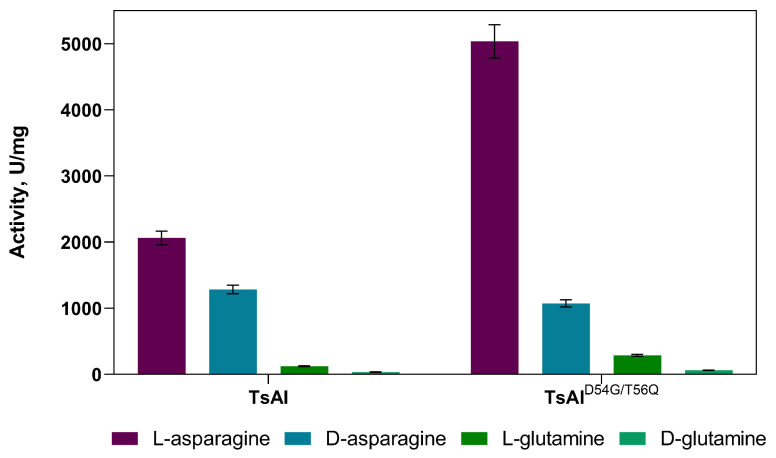
Substrate specificity of the TsAI variants.

An increase in the specific L-asparaginase activity of the TsAI^D54G/T56Q^ mutant compared with the wild-type TsAI was shown in our previous study [10]. To decipher the substrate specificity of both forms, activity towards D-asparagine and L- and D-glutamine was determined (Figure 1).

If the activity towards the natural substrate L-asparagine is set as 100%, recombinant thermo-ASNases exhibit extremely high hydrolysis activity towards D-asparagine compared with 1–6% for mesophilic L-ASNases [18,19,20]. Relative activity of 62% and 21% to D-asparagine was observed for TsAI and TsAI^D54G/T56Q^, respectively. Minor differences were detected in L-glutaminase activity—4.5÷5.7% of the activity with L-asparagine for both enzymes. Trace D-glutaminase activity was observed for TsAI and TsAI^D54G/T56Q^ (Figure 1).

#### 2.1.2. Kinetic Characteristics

D54G and T56Q mutations are located in close proximity to the enzyme–substrate interaction site. The influence of these substitutions on the kinetic parameters of substrate hydrolysis was assessed (Table 1, Supplementary Materials, Figures S1–S4).

For the mutant form, minor no statistically significant differences were found in the values of K_m_ (increase) and V_max_ (decrease) for D-asparagine and L- and D-glutamine. However, for the natural substrate L-asparagine, a two-fold increase in the K_m_ and V_max_ values was observed for the mutant. This may indicate that the mutations D54G and T56Q reduce the binding affinity of TsAI for L-asparagine and simultaneously enhance the catalytic power of its hydrolysis.

### 2.2. Overall Crystal Structure of the Wild-Type TsAI and Structural Comparison with Type I and Type II L-Asparaginases

**Figure 2:**
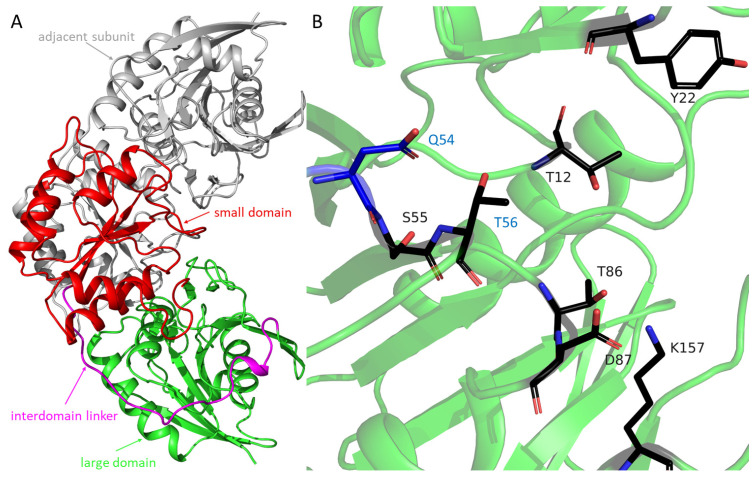
TsAI structure. (**A**) Functional dimer of TsAI. The small domain is colored in red, the large domain in green, and the interdomain linker in magenta. The adjacent subunit is colored in gray. (**B**) Residues of the active site are shown as black stick model and labeled. The residues Q54 (shown as blue sticks) and T56 selected for mutation are labeled in blue.

The crystal structure of TsAI was solved at 1.9 Å. The asymmetric unit contained one copy of the protein. Crystal contact analysis showed that TsAI is a dimer in crystal (Figure 2A) as in solution. The subunit of TsAI consists of two α/β domains: an N-terminal large domain (residues 1–184, consisting of twelve mixed β-sheets and four α-helices, Figure 2A,B) and a C-terminal small domain (residues 207–331, consisting of three β-sheets and five α-helices) connected by an interdomain linker (residues 185–206).

In the N-terminal domain, the active site pocket of TsAI includes residuesthat are highly conserved among all types of L-ASNases: Y22, S55, T56, T86, D87, K157, and T12, which initiates the reaction (Figure 2B). TsAI acts as a hydrolase, using weak nucleophile T12 to attack the substrate.

TsAI shares 63% amino acid identity with the thermo-ASNase *Thermococcus kodakarensis* TkA (PDB ID 5OT0) (Table 2), the only enzyme crystallized from highly active L-ASNases of *Thermococcus* sp. The identity of TsAI and two crystallized to date low-active thermo-ASNases from *Pyrococcus furiosus* (PfA, PDB ID 4Q0M) and *Pyrococcus horikoshii* (PhA, PDB ID 1WLS) is 56% (Table 2). TsAI has only 33% and 34% amino acid identity with the mesophilic bacterial L-ASNases from *E.coli* EcAI (PDB ID 2P2D) and EcAII (PDB ID 6UOD) (Table 2), respectively, that are considered as a reference for structural comparison.

**Figure 3:**
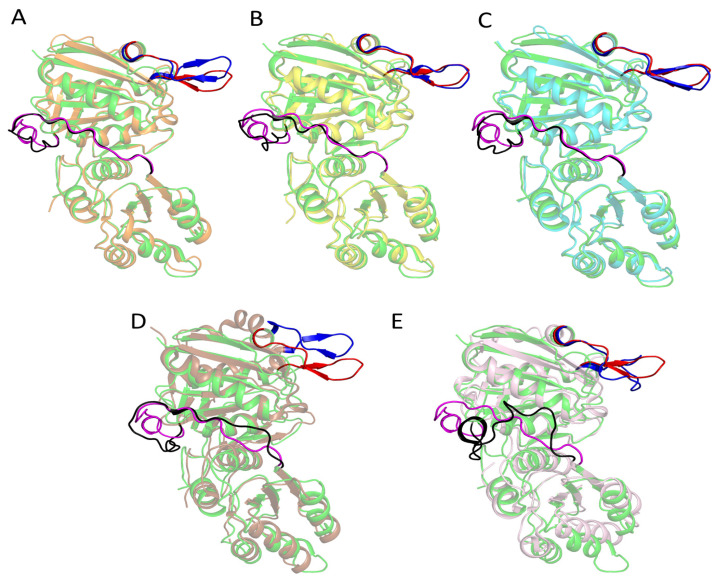
Structural superposition of TsAI (green) with L-asparaginases from hyperthermophilic archaea-TkA (**A**), PhA (**B**), PfA (**C**), and mesophilic bacteria EcAI (**D**), EcAII (**E**). TkA, PhA, PfA, EcAI, and EcAII are shown in orange, yellow, cyan, brown, and pink, respectively. Gatekeeper is shown as red in TsAI (residues 14–29) and blue in TkA (13–28), PhA (13–28), PfA (13–28), EcAI (17–30), and EcAII (14–33). Interdomain linker is shown as magenta in TsAI (residues 185–206) and grey in TkA (184–203), PhA (182–201), PfA (182–201), EcAI (190–211), and EcAII (190–213). Other parts of structures are shown semitransparent for clarity.

Structural superposition of TsAI with TkA, PhA, PfA, EcAI, and EcAII (RMSD between TsAI and these structures are 0.5 Å, 0.6 Å, 0.5 Å, 1.5 Å, and 1.5 Å, respectively) revealed high conservation of the active site architecture and similarity in overall topology of non-catalytic regions, regardless of type, origin (taxonomy), and degree of sequence identity (Figure 3). However, minor structural changes in various regions, especially those adjacent to the active site, significantly affect the functioning and biochemical properties of these enzymes. The main differences in the structures of L-ASNases included different conformations of flexible loops (14–29, 175–183, 236–240, numbering according to TsAI), C-terminal tail, and linker between domains. Key regions in the structures of L-ASNases that are not well superimposed are described below.

L-ASNases have a flexible lid that covers the active site (Figure 3). This flexible loop, which forms a so-called gatekeeper for substrate entry, has the same length in TsAI (residues 14–29) as in TkA (residues 13–28), PhA (13–28), and PfA (13–28), longer than in EcAI (17–30), and shorter than in EcAII (14–33). The structure restricts/provides access to the active site of the enzyme and plays a key role in catalysis, affecting its dynamic properties [23].

The C-terminal domain is reported to play an important role in structure stabilization [16,25]. The C-terminal tail of TsAI contains 9 a.a. (residues 323-331), archaeal TkA—9 a.a. (320-328), PhA—11 a.a. (318-328), mesophilic bacterial EcAI—11 a.a. (327-337), and EcAII—2 a.a. (325-326). It is assumed that variability in the length of the C-terminal tail affects the stability of L-ASNases, in particular, their thermostability [8,16]. Thus, deletion of 9 extra residues, if compared with mesophilic L-ASNases, led to a significant decrease in the thermostability of truncated archaeal thermo-ASNase from *P. yayanosii* [16]. TsAI is 7 aa longer than EcAII, including conserved thermo-ASNases Q328, involved in a network of interactions with the backbone oxygen atom of T323 and nitrogen atoms of Y325 and V326, as well as the side chain of K303.

The interdomain linker is one of the variable elements with an unclear role in the stability and activity of L-ASNases. The loop connects protein domains and is variable in both sequence and length. The linker of TsAI (residues 185–206, 22 a.a.) is longer than in TkA (184–203, 20 a.a.), PfA (182–201, 20 a.a.), and PhA (182–201, 20 a.a.); has the same length as in EcAI (190–211, 22 a.a.); and is shorter than in EcAII (190–213, 24 a.a). Attempts to determine the role of the linker in maintaining activity were performed using the PfA enzyme. It was revealed that separate domains of PfA retain activity in the absence of the loop [23]. Nevertheless, further deletion of adjacent β-strand residues in the N-terminal domain suggests that the region is essential for stability at elevated temperatures [26].

Thus, L-ASNases displaying the same fold exhibit differences in the spatial position of the flexible regions.

### 2.3. Structural Differences Between Wild-Type TsAI and Mutant TsA^ID54G/T56Q^ That Affect Their Activity and Stability

**Figure 4:**
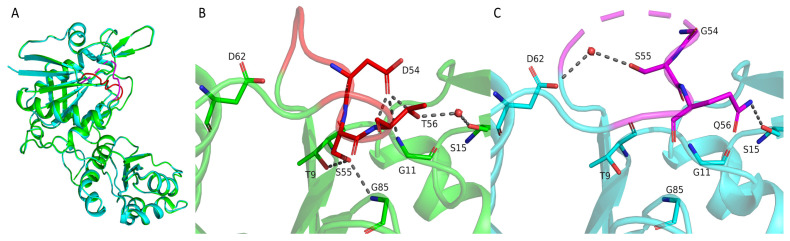
Comparison of TsAI structures. (**A**) Superposition of TsAI (green) and TsAI^D54G/T56Q^ (cyan). Loop M51-L57 is colored in red and magenta in TsAI and TsAI^D54G/T56Q^, respectively. (**B**) The loop M51-L57 region in the wild-type enzyme TsAI. (**C**) The loop M51-L57 region in the mutant TsAI^D54G/T56Q^. Polar contacts are shown as grey dotted lines. Water molecules are shown as red spheres. For clarity, only the residues described in the text are shown.

Superposition of TsAI and its mutant form TsAI^D54G/T56Q^ showed stability of the fold for these structures (the RMSD for the Cα atoms does not exceed 0.2 Å, Figure 4). The most significant difference between them was observed in the conformation of residues 51–57, where two substitutions occurred. In the TsAI structure, T56 forms a water-mediated hydrogen bond with S15. The side chain of D54 forms a hydrogen bond network with G11, S55, and T56. The position of this loop is additionally fixed by the interaction of the side chain S55 with T9 and G85. The point mutation T56Q led to the formation of a hydrogen bond between Q56 and S15 side chains in TsAI^D54G/T56Q^. At the same time, the D54G mutation destroyed the network of hydrogen bonds. Together, this led to a change in the conformation of this loop that was additionally fixed by the formation of a water-mediated hydrogen bond between the S55 and D62 side chains. Moreover, the part of this loop (residues 52 and 53) has no electron density, confirming the increased mobility of this region.

Overall, the experimental data show that the increased flexibility of the loop M51-L57 due to mesophilic-like replacement D54G/T56Q is the key structural difference between TsAI forms. The loop is located in close proximity to the active site of the enzyme affecting its conformational flexibility. The significance of loop mobility for TsAI enzymatic activity is confirmed by the two-fold increase in activity in the mutant form after disruption of “redundant” hydrogen bonds [10].

On the other hand, TsAI^D54G/T56Q^ exhibits a sharper temperature optimum curve and increased time-dependent loss of activity compared with the wild-type TsAI [10]. Thus, the higher rigidity of the loop M51-L57 is significant for maintaining increased thermostability of the native thermo-ASNase TsAI. In the wild-type enzyme, adaptive mutations stabilized the loop by a network of H-bonds and replaced the highly thermolabile glutamine residue with threonine to ensure proper function at elevated temperatures.

### 2.4. Elucidation of Substrate-Enzyme Interaction and Stereoselectivity of TsAI Forms Using Molecular Docking Analysis

**Figure 5:**
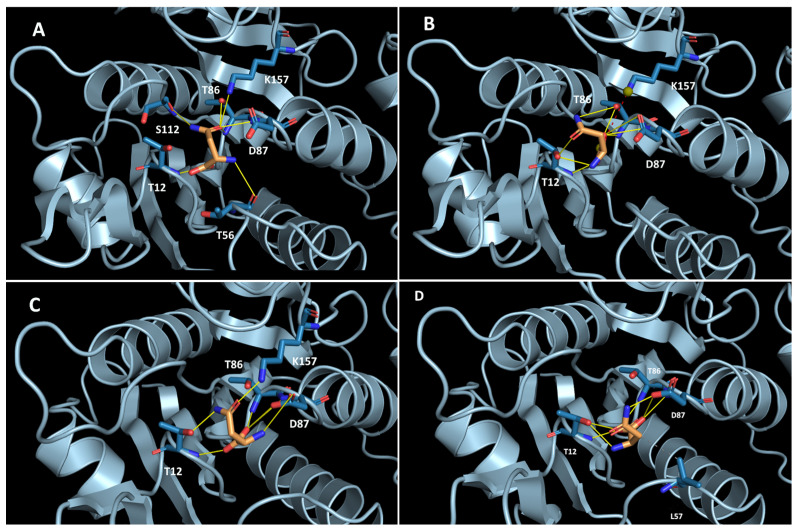
Designed complex of thermo-asparaginase TsAI and the mutant TsAI^D54G/T56Q^ with asparagine enantiomers: (**A**) TsAI interaction with L-asparagine; (**B**) TsAI interaction with D-asparagine; (**C**) TsAI^D54G/T56Q^ interaction with L-asparagine; (**D**) TsAI^D54G/T56Q^ interaction with D-asparagine. H-bonds are shown as yellow lines, salt bridge as red dashed lines, and hydrophobic interactions in grey dashed lines.

TsAI displays exceptionally high D-asparaginase activity. Mesophilic-like substitutions induced changes in the enzyme stereospecificity. Unfortunately, attempts to obtain TsAI crystals with asparagine enantiomers were not successful. To gain insight into the substrate-enzyme interaction and enantioselectivity of TsAI forms, molecular docking was performed. The results showed that L-asparagine interacts in the active site of TsAI with Thr12, Thr56, Thr86, Asp87, Ser112, and Lys157 by forming H-bonds (Figure 5A). Carbonyl oxygen is located in the hollow where it can form H-bonds with four residues: T86, D87, S112, and K157. Docking of D-asparagine in the active site of TsAI predicted that the D-asparagine position is shifted and the oxygen of the carboxyl group is accommodated hollow where the carbonyl oxygen of L-asparagine is located. The amide group of D-asparagine is turned in the opposite direction. It lost interactions with T56 and S112 and formed an H-bond with T86 (Figure 5B). Thus, according to docking analysis, D-asparagine orientation in the TsAI active site is not optimal for catalysis, which explains the slow rate of its hydrolysis.

Docking of L-asparagine in the active site of TsAI^D54G/T56Q^ revealed that its position is similar to that found for the wild-type TsAI (Figure 5C). However, local structural changes occurred in the loop M51-L57 containing mutated residues in the TsAI^D54G/T56Q^. This resulted in disruption of L-asparagine amine nitrogen interaction with the backbone of this loop. High L-asparagine mobility in the active site increased the K_m_ value in the mutant form. The position of D-asparagine in the TsAI^D54G/T56Q^ active site was similar to its position in the wild-type enzyme.

## 3. Discussion

Since the first crystallized L-ASNase EcAII in 1993, efforts have been performed primarily to determine the structures of tetrameric EcAII-related L-ASNases from mesophilic bacterial hosts. To date, structures of three archaeal thermo-ASNases (TkA, PhA, PfA) occupying an intermediate position between typical type I and type II L-ASNases were available [15,22,23]. Two of them belong to relatively low-active thermo-ASNases from *Pyrococcus* sp. [8]. Structural data about promising for biotechnology thermo-ASNases from *Thermococcus* sp. was limited by TkA.

In this study, the structure of TsAI was solved at a high resolution of 1.9 Å. Superposition of the TsAI structure with hyperthermophilic archaeal TkA, PfA, and PhA and mesophilic bacterial EcAI and EcAII revealed high conservation of the active site architecture and similarity in overall topology of non-catalytic regions, regardless of the type, origin, and degree of sequence identity. However, differences, even minor, in the spatial position of flexible regions, such as gatekeeper (TsAI, 14–29) and interdomain linker TsAI (TsA, 185–206), cause significant differences in the functioning and biochemical properties of these enzymes. This is confirmed by the point mutations replacing thermo-ASNase amino acid residues with mesophilic ones occupying the corresponding positions in these regions.

### 3.1. Engineering of Improved L-ASNase Variants Using Nature-Inspired Mutagenesis

From sequence alignment and structure–mutational analysis, the residues that are highly conserved within the thermo-ASNase and mesophilic L-ASNase groups but differ between them can represent mutational hotspots. In particular, they affect the rigidity and flexibility characteristics of regions neighboring an active site and play a key role in efficient functioning.

In this study, the mesophilic-like triad GSQ was restored in the mutant form of thermo-ASNase *T.sibiricus*. The mesophilic-like substitution in close proximity to the active site increased activity more than two-fold at the optimum temperature of 90 °C [10]. The structure of TsAI^D54G/T56Q^ solved in this study revealed structural differences underlying the increase in activity in the mutant form. The network of hydrogen bonds formed between D54 and G11, S55, and T56, along with the interaction of the side chain of S55 with T9 and G85, was shown to restrict loop motion in the wild-type enzyme. According to structural data, the mesophilic-like mutations D54G/T56Q destroyed the network of hydrogen bonds and altered neighbor interactions of the substrate-binding S55. This resulted in increased flexibility and a change in conformation of the loop M51-L57, located in close proximity to the active site of the enzyme.

The same approach was successfully applied for engineering thermo-ASNase *P. furiosus* PfA. In PfA, the residue K274 strongly interacting with the neighboring β-hairpin loop was replaced by glutamate residue found at the corresponding position of the mesophilic EcAII. Disruption of excessive interactions that stabilized the loop and restricted substrate access resulted in increased catalytic efficiency and a six-fold decrease in the K_m_ value at 80 °C [27].

Mesophilic-like mutations focused on residues adjacent to or in close proximity to the active site of the thermo-ASNase *T.kodakarensis* TkA—T17G, K299L—exhibited a 15–20% increase in activity [28]. It is suggested that one polar contact in the mutant TkA^T17G^ instead of four in the wild-type enzyme facilitated substrate binding and product release due to increased flexibility. Disruption of 4 of the 6 polar contacts in the mutant TkA^K299L^ increased activity due to higher loop 319-328 flexibility, which facilitated lid movement.

Similar results were obtained after mesophilic-like substitution S17G in the loop around the active site of the thermo-ASNase *Pyrococcus yayanosii*. A 1.7-fold increase in enzymatic activity was observed [29].

Thus, contacts between residues in the moving and active site neighboring regions must be flexible enough to provide these movements. Mesophilic-like substitutions in thermo-ASNases in these regions allow maintaining the efficient ratio of rigidity/local flexibility for enzyme activity.

Vice versa, thermophilic-like mutations allow improving characteristics of mesophilic L-asparaginases. Replacement of residues of L-ASNases *E.coli* EcAII and *Bacillus subtilis* BsAII with corresponding thermophilic ones in the flexible regions adjacent to the active site—EcAII^G57D^, EcAII^L305K^, BsAII^L354K^, BsAII^G107D^—improved their thermostability and, in some cases, increased activity [16,30].

### 3.2. The Flexibility of the Loop M51-L57 (TsAI) Is Essential for Thermo-ASNases Activity

The loop M51-L57 is located in close proximity to the active site of the TsAI enzyme and affects its conformational flexibility. Structural superposition exhibited similarity of this region among thermo-ASNases. The four-membered DSTL motif of the loop is highly conserved for these enzymes. In a recent study, the importance of loop flexibility was confirmed using another thermo-ASNase from *T. kodakarensis* TkA [28]. At position 4 of the DSTL motif, the leucine residue in TkA was replaced by aspartate, found at the corresponding position of the mesophilic EcAII. The TkA^L56D^ mutant formed additional polar contacts compared with the wild-type enzyme. The mutant displayed the same optimum temperature of 85 °C as the wild-type enzyme, high thermostability, and a significant decrease in activity at 85 °C [28]. This is supposed to be attributed to the restriction of loop mobility that led to decreased structural flexibility and activity at the optimum temperature.

Thus, the flexibility of the loop M51-L57, numbered according to TsAI appears to be essential for thermo-ASNase activity. The L/D substitution in the highly conserved DSTL motif impairing loop movement can decrease thermo-ASNase activity. D/G and T/Q mutations at positions 1 and 3 of the DSTL motif restore the GSQ triad characteristic of a number of mesophilic homologues, including EcAII, facilitating loop mobility and increasing activity. This can be applied for improving properties of the wide range of thermophilic L-asparaginases.

### 3.3. Thermo-ASNase Features in Substrate Specificity: Biological Significance and Practical Importance

L-ASNases are known for their ability to catalyze conversion of structurally related substrates. These enzymes hydrolyze not only L-asparagine but also its enantiomer D-asparagine [19,21,31]. L-ASNases can display at least trace L-glutaminase [18,32,33] and even D-glutaminase activity [18,33]. Competition between substrates for the active site has been reported in studies of various L-ASNases [18,33,34,35]. Thus, if highly abundant, the less preferred L-glutamine can outcompete L-ASNase’s natural substrate.

TsAI displays relatively low L-glutaminase activity (~5% of the activity with L-asparagine) and extremely high D-asparaginase activity. The relative activity of TsAI towards D-asparagine is up to 60% compared with 1–6% for mesophilic L-ASNases [18,19,20].

Unfortunately, attempts to obtain TsAI crystals binding with substrates were not successful. Molecular docking studies showed that, in contrast to the complex with L-asparagine, D-asparagine orientation in the TsAI active site is not optimal for catalysis. Previously, in a study by Aghaiypour et al., the crystal structure of bacterial type II L-ASNase from *E. chrysanthemi* ErAII was solved in complex with D-Asp (PDB ID: 1HG1) [36]. Significant differences were observed between ErAII-L-aspartate and ErAII-D-aspartate complexes. It was found that the inverted stereochemistry of the D-enantiomer causes the side chain to orient towards the flexible N-terminal loop. This impairs proper ordering and closure of the flexible N-terminal loop, preventing the formation of a stable active conformation [36].

However, thermo-ASNases exhibit much higher relative D-asparaginase activity compared with their mesophilic counterparts [18,19,20]. High D-asparaginase activity was also reported for hyperthermophilic TkA (optimum temperature 85 °C)—50% compared with the L-asparaginase activity [21] L-asparaginase from *Mycobacterium gordonae* (GmASNase) (optimum temperature 50 °C)—22% [37].

Citri et al. revealed an increase in the relative activity of mesophilic EcAII toward D-asparagine upon heating [19,38]. Partial thermal disruption of the enzyme caused transient changes in stereospecificity in favor of D-asparagine compared with normal conditions. The observed shifts in stereospecificity were reversible. Thus, it can be supposed that high working temperatures can induce specific conformational changes in thermo-ASNases, reducing discrimination towards the D-enantiomer of asparagine.

Indeed, molecular docking of crystallized structures revealed no changes in D-asparagine interaction between TsAI forms. However, these results do not take into account the special conditions of thermo-ASNase functioning. The differences in the substrate specificity were observed under high temperatures. Previously, fluorescence spectroscopy showed that TsAI undergoes changes in the tertiary structure under heating. In order to exhibit unique thermophilic behavior towards substrates, in particular, high specific activity, TsAI needs to acquire a more extended conformation, which is achieved only at temperatures above 80 °C [39]. Upon heating, the relative D-asparaginase activity of TsAI reached 60%, which is consistent with high D-asparaginase activity reported for other thermophilic L-ASNases [21,37]. In the TsAI^D54G/T56Q^ mutant, mesophilic-like substitutions affected the active site, which became similar to the active site configuration of the EcAII enzyme. As a consequence, a shift in the enantioselectivity of the mutant was observed; it began to resemble a mesophilic enzyme with moderate activity towards D-asparagine.

Though the biological significance of the high D-asparaginase activity of thermo-ASNases is still unclear, it was reported that in cells of *Pyrococcus* sp. and *Thermococcus* sp., the D-aspartate content can reach more than 40% of the total aspartic acid [40]. Moreover, aspartate racemases, converting L-aspartate to D-enantiomer, are widely distributed among these hyperthermophiles. This can be an adaptive feature for surviving at high temperatures. Racemization of amino acids proceeds slowly under normal conditions: 3500 years for aspartate at 25 °C. But the reaction is greatly accelerated at high temperatures of about 100 °C: 20% of L-aspartate is converted to the D-enantiomer in 1 day at 106.5 °C [41]. The ability to utilize D-asparagine might be beneficial in such conditions. It is also assumed that thermo-ASNases act in concert with other enzymes involved in the metabolism of D-amino acids, where D-aspartate (D-asparagine) plays a key role [42,43].

The ligand-binding preference of a particular L-ASNase is one of the most important characteristics that determine its application. For example, in clinical practice, it is assumed that toxic side effects of L-ASNase treatment are associated with residual glutaminase co-activity [44,45,46]. So, numerous efforts were performed either to engineer or discover new glutaminase-free variants [47,48,49,50]. While the main attention was paid to the study of glutaminase activity, it was believed that D-asparagine hydrolysis by L-ASNases has no significant value—neither functional nor practical. However, for thermo-ASNases, the ability to hydrolyze D-asparagine can be important for efficient functioning in extreme temperature conditions.

Moreover, the observed high D-asparaginase co-activity can be considered as an additional advantage of thermo-ASNases for food industry application, since both L- and D-asparagine in foods are effectively converted in the non-enzymatic Maillard reaction to toxic derivatives under processing [51].

### 3.4. The Potential Implications of Research Outcomes

The results of the study provide details about the structure and specificity of TsAI variants—the wild-type and the mutant with increased activity—that are promising for use in biotechnology. The obtained data confirm that the structural flexibility of the regions neighboring the active site is a prerequisite for high enzymatic activity. Mobility of the loop M51-L57 is essential in maintaining thermo-ASNase activity. Due to the structural similarity of thermo-ASNases, the approaches summarized can be applied for engineering a wide range of these enzymes.

Structural data of thermo-ASNases TsAI provide a basis for further enzyme improvement. The enzyme was shown to be a promising biocatalyst for biotechnology, in particular, the food industry [51,52]. In our previous study, it was revealed that TsAI, by hydrolyzing asparagine, a substrate for acrylamide synthesis, reduces the content of this toxic derivative in multicomponent food mixtures by up to 98% [51,52]. Improved TsAI variants with high activity and stability under food-processing conditions are perspective for use in high-temperature food technologies.

## 4. Materials and Methods

### 4.1. Protein Expression and Purification

L-ASNase TsAI (GenBank accession No. WP_015849943.1) was heterologously expressed in *E. coli* cells using synthetic gene *tsA*_mod (GenBank accession No. MW981255) inserted into the pET-28a(+) vector. TsAI^D54G/T56Q^ mutant was designed and constructed as previously described [10].

Recombinant strains harboring constructed plasmids expressing native and mutant forms of TsAI were grown at 37 °C in the media containing kanamycin (0.05 mg/mL) as previously described [9]. Purification of the enzymes was carried out at 4 °C according to the procedure described in our previous study [9]. The target proteins were analyzed by SDS-PAGE, frozen, and stored at −20 °C.

### 4.2. Measurement of Enzyme Activity and Kinetic Parameters

The activity of L-ASNases was determined by direct Nesslerization [53,54]. The reactions were performed at 90 °C in Tris–HCl buffer (0.05 M, pH 9.0) for TsAI and TsAI double mutant. Specific enzyme activity was expressed in U/mg protein.

Determination of kinetic parameters (K_m_ and V_max_) of TsAI and TsAI^D54G/T56Q^ [55] was performed as previously described [9] by subsequent incubation of the enzymes in 0.25–20 mM L-asparagine, 0.25–30 mM L-glutamine or D-asparagine, or 0.25–40 mM D-glutamine.

### 4.3. Crystallization, Data Collection, and Structure Refinement

The enzymes were concentrated in 10 mM Tris-HCl (pH 6.5) with Spin-X UF centrifugal concentrators 10k MWCO (Corning, NY, USA). Initial crystallization screening was performed on a robotic system (Rigaku Americas Corporation, The Woodlands, TX, USA) using 96-well VDX plates (Hampton Research, Aliso Viejo, CA, USA) and commercial crystallization screens from Hampton Research (Aliso Viejo, CA, USA) and Molecular Dimensions Inc. (Holland, OH, USA) by the “hanging drop” vapor diffusion method. A 10 mg/mL of the TsAI and its mutant form TsAI^D54G/T56Q^ in Tris-HCl buffer pH 6.5 were mixed with the crystallization solution in the ratios 1:1 (0.2 μL drop volume), 2:1, and 1:2 (0.3 μL drop volume). The volume of the precipitant solution in the reservoir was 50 µL. The initial crystallization hit for TsAI was observed under the following conditions: 0.2 M ammonium acetate, 20% *w*/*v* polyethylene glycol 3350. For TsA^D54G/T56Q^: 0.8 M lithium sulfate monohydrate, 0.1 M sodium acetate trihydrate pH 4.0, and 4% *v*/*v* polyethylene glycol 200. Further optimization of crystal growth was made using the “hanging drop” vapor-diffusion method in 24-well VDX plates (Hampton Research). The drop volume was increased to 3 μL, and the volume of the precipitant solution to 500 μL. Crystals of TsAI and TsAI^D54G/T56Q^ were briefly soaked in a mother liquor containing 20% glycerol immediately before diffraction data collection and flash-frozen in liquid nitrogen. Datasets were collected at 100K at Rigaku OD XtaLAB Synergy-S. The datasets were indexed, integrated, and scaled using the CrysAlisPro software v.1.0.43 (Oxford Diffraction/Agilent Technologies UK Ltd., Yarnton, UK). Space groups were suggested by Pointless [47] as P22_1_2_1_ for both structures (Table 3).

The structure of the TsAI was solved by the molecular replacement method using the MOLREP program v. 11.7. [48] with the atomic coordinates of the thermo-ASNase from *Thermococcus kodakarensis* (TkA; PDB ID 5OT0) as a starting model, while the structure of the TsAI^D54G/T56Q^ was solved using the wild-type TsAI structure. One copy of the protein was found in an asymmetric unit of both structures. The refinement of all structures was carried out using the REFMAC5 program of the CCP4 suite [49]. The visual inspection of electron density maps and the manual rebuilding of the model were carried out using the COOT interactive graphics program [50]. The isotropic B-factor and the hydrogen atoms in fixed positions were included during the refinement. For TsAI^D54G/T56Q^, TLS was introduced during the final refinement cycles.

The visual inspection of the modeled structure was carried out using the COOT program and the PyMOL Molecular Graphics System, Version 4.6 (Schrödinger, Portland, OR, USA). The structure comparison and superposition were made using the PDBeFOLD program v. 2.58 [51]. The contacts were analyzed using the PDBePISA v. 1.48 [52].

### 4.4. Molecular Docking

Structures of L-asparagine and D-asparagine were generated using the SYBYL 8.1 program (Tripos Inc., St. Louis, MO, USA). Structures of the compounds and L-asparaginase were optimized by energy minimization using the Tripos force field in a vacuum. The partial atomic charges were calculated by the Gasteiger–Huckel method. Docking was carried out by Vina Autodock v. 1.2.0 [56]. The AutoDock Tools package was used to set docking parameters and prepare structures of protein and ligands. The binding site of TsAI was determined by the position of aspartate from the structure of its complex with asparaginase from *E.coli* (PDBid 3eca) after spatial alignment of proteins. The value of exhaustiveness was 128. The ligand positions obtained from docking were ranked and chosen based on binding energies and geometrical properties. Analysis of intermolecular interactions in protein–ligand complexes was performed using the PLIP server [57].

## Figures and Tables

**Table 1:** Kinetic parameters of TsAI and its mutant form.

Substrate	K_m_, mM		V_max_, mM/min	
	TsAI	TsAI^D54G/T56Q^	TsAI	TsAI^D54G/T56Q^
L-asparagine	3.10 ± 0.15	6.03 ± 0.15	4.02 ± 0.38	8.17 ± 0.24
D-asparagine	8.63 ± 0.48	9.18 ± 0.86	0.66 ± 0.15	0.59 ± 0.12
L-glutamine	11.09 ± 0.25	11.41 ± 0.27	0.28 ± 0.09	0.25 ± 0.06
D-glutamine	13.86 ± 0.86	14.40 ± 1.60	0.18 ± 0.03	0.18 ± 0.04

**Table 2:** List of L-asparaginases used for analyses and their main characteristics.

Enzyme	Organism	PDB ID	Sequence Length	Type /Localization	Oligomeric State	Specific Activity, U/mg/Topt, °C	Refs.
**Hyperthermophilic L-asparaginases**							
TsAI	*Thermococcus sibiricus*	9UFQ	331	I-like/cytosolic	Homodimer	2066/90	[10]
TsAI^D54G/T56Q^	*Thermococcus sibiricus*	9UFR	331	I-like/cytosolic	Homodimer	5038/90	[10]
TkA	*Thermococcus kodakarensis*	5OT0	328	I-like/cytosolic	Homodimer	2350/85	[21,22]
PhA	*Pyrococcus horikoshii*	1WLS	328	I-like/cytosolic	Homodimer	No data	[15]
PfA	*Pyrococcus furiosus*	4Q0M	327	I-like/cytosolic	Homodimer	330/80	[23]
**Mesophilic L-asparaginases**							
EcAI	*Escherichia coli*	2P2D	358	I type/cytosolic	Homotetramer		[24]
EcAII	*Escherichia coli*	6UOG	326	II type/periplasmic	Homotetramer	91/37	

**Table 3:** Data collection, processing, and refinement statistics.

	TsAI	TsAI^D54G/T56Q^
**Data collection**		
Diffraction source	Institute of Organic Chemistry RAS (Rigaku OD XtaLAB Synergy-S)	
Wavelength (Å)	1.54	
Temperature (K)	100	
Detector	HyPix-6000HE	
Crystal-to-detector distance (mm)	37	38
Rotation range per image (°)	0.15	0.4
Total rotation range (°)	300	360
Space group	P22_1_2_1_	
*a*, *b*, *c* (Å)	45.19, 57.79, 131.16	45.01, 57.45, 130.65
α, β, γ (°)	90.00, 90.00, 90.00	90.00, 90.00, 90.00
Average mosaicity (°)	0.89	1.25
Resolution range (Å)	24.11–1.90 (1.94–1.90)	22.70–1.90 (1.94–1.90)
Completeness (%)	95.3 (92.8)	99.4 (96.3)
Average multiplicity	11.2 (13.2)	12.0 (8.2)
〈*I*/σ(*I*)〉	21.7 (7.8)	31.5 (5.0)
Rmeas (%)	8.4 (32.1)	8.7 (42.5)
CC_1/2_	99.9 (97.5)	99.9 (93.7)
**Refinement**		
*R_fact_ (%)*	16.7	17.5
*R_free_ (%)*	21.4	22.5
RMSD Bonds (Å)	0.01	0.02
RMSD Angles (°)	1.80	2.15
**Ramachandran plot**		
Ramachandran favoured (%)	97.0	96.9
Ramachandran allowed (%)	2.4	2.5
PDB ID	9UFQ	9UFR

## Data Availability

The data presented in this study are contained within the article.

## Supplementary Materials

The following supporting information can be downloaded at: https://www.mdpi.com/article/10.3390/ijms26125437/s1.
