# Supplementary material for: Hyperthermophilic L-Asparaginase from Thermococcus sibiricus and Its Double Mutant with Increased Activity: Insights into Substrate Specificity and Structure
Source: Int J Mol Sci. 2025 Jun 6;26(12):5437. doi: 10.3390/ijms26125437 (PMC12193700; doi:10.3390/ijms26125437)
Supplement: Supplementary file 1 [file ijms-26-05437-s001.zip › ijms-3627650-supplementary.pdf]

Supplementary Materials

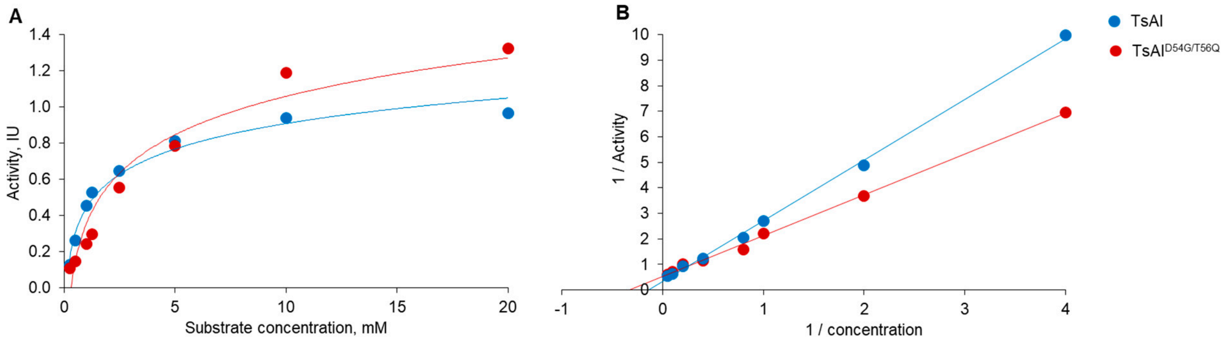

Figure S1. Graphs of kinetic curves for determining the kinetic parameters of enzymes for L-asparagine. The graphs were obtained using 0.04 mg of purified enzyme. (**A**) The graph of the dependence of enzymatic activity on L-asparagine concentration. (**B**) Double-reciprocal Lineweaver-Burk plot.

\

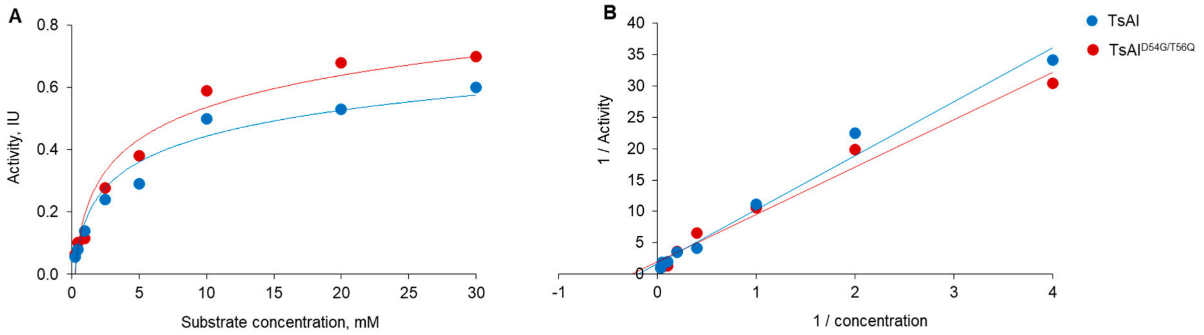

Figure S2. Graphs of kinetic curves for determining the kinetic parameters of enzymes for D-asparagine. The graphs were obtained using 0.1 mg of purified enzyme. (**A**) The graph of the dependence of enzymatic activity on L-asparagine concentration. (**B)** Double-reciprocal Lineweaver-Burk plot.

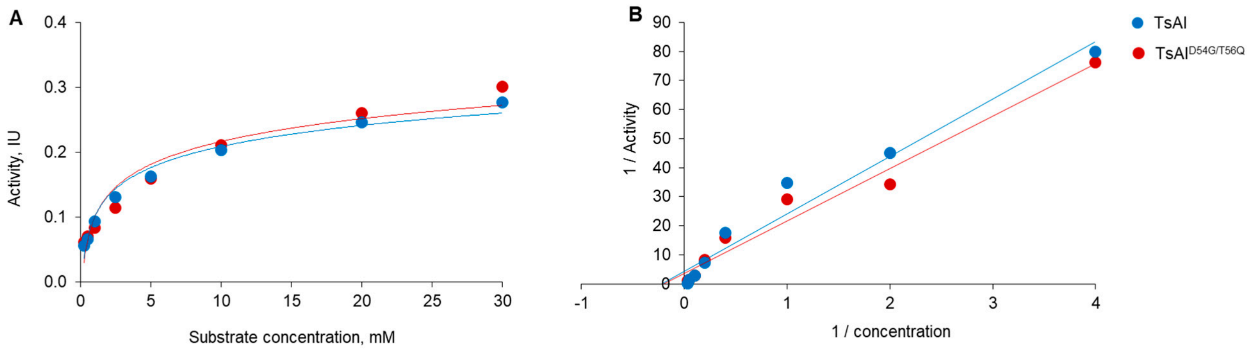

Figure S3. Graphs of kinetic curves for determining the kinetic parameters of enzymes for L-glutamine. The graphs were obtained using 0.1 mg of purified enzyme. (**A**) The graph of the dependence of enzymatic activity on L-asparagine concentration. (**B**) Double-reciprocal Lineweaver-Burk plot.

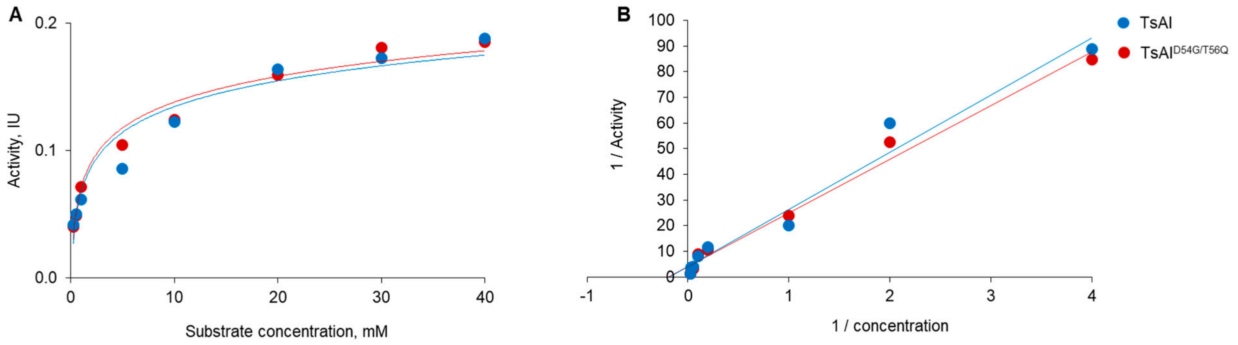

Figure S4. Graphs of kinetic curves for determining the kinetic parameters of enzymes for D-glutamine. The graphs were obtained using 0.1 mg of purified enzyme. (**A**) The graph of the dependence of enzymatic activity on L-asparagine concentration. (**B**) Double-reciprocal Lineweaver-Burk plot.
